# AMPK activation by Tanshinone IIA protects neuronal cells from oxygen-glucose deprivation

**DOI:** 10.18632/oncotarget.23391

**Published:** 2017-12-17

**Authors:** Yingfeng Weng, Jixian Lin, Hui Liu, Hui Wu, Zhimin Yan, Jing Zhao

**Affiliations:** ^1^ Department of Neurology, Minhang Branch, Zhongshan Hospital, Fudan University, Shanghai, China

**Keywords:** OGDR, neuroprotection, Tanshinone IIA, AMP-activated protein kinase (AMPK), Ppm1e

## Abstract

The current study tested the potential neuroprotective function of Tanshinone IIA (ThIIA) in neuronal cells with oxygen-glucose deprivation (ODG) and re-oxygenation (OGDR). In SH-SY5Y neuronal cells and primary murine cortical neurons, ThIIA pre-treatment attenuated OGDR-induced viability reduction and apoptosis. Further, OGDR-induced mitochondrial depolarization, reactive oxygen species production, lipid peroxidation and DNA damages in neuronal cells were significantly attenuated by ThIIA. ThIIA activated AMP-activated protein kinase (AMPK) signaling, which was essential for neuroprotection against OGDR. AMPKα1 knockdown or complete knockout in SH-SY5Y cells abolished ThIIA-induced AMPK activation and neuroprotection against OGDR. Further studies found that ThIIA up-regulated microRNA-135b to downregulate the AMPK phosphatase Ppm1e. Notably, knockdown of Ppm1e by targeted shRNA or forced microRNA-135b expression also activated AMPK and protected SH-SY5Y cells from OGDR. Together, AMPK activation by ThIIA protects neuronal cells from OGDR. microRNA-135b-mediated silence of Ppm1e could be the key mechanism of AMPK activation by ThIIA.

## INTRODUCTION

Ischemia-reperfusion shall cause damages to neurons [1, 2]. In cultured neurons, oxygen-glucose deprivation (ODG) and re-oxygenation (OGDR) was applied to mimic the ischemia-reperfusion injuries [3–6]. Tanshinone IIA (ThIIA) is a phenanthrenequinone derivative from the Traditional Chinese Medicine Danshen [7, 8]. ThIIA has been utilized for the treatment of many diseases [7, 8]. The potential neuroprotective effect of ThIIA against OGDR is largely unknown.

Maintaining the physiological energy level in the neurons is vital for key cerebral behaviors. AMP-activated protein kinase (AMPK) is a key energy sensor [9–12]. Existing literatures have indicated that physiological AMPK also participates in brain development, neuronal polarization and other neuronal activities [9–12]. Additionally, deregulation of AMPK signaling could be involved in the development of neurodegenerative diseases [9–12]. Therefore, AMPK is emerging as a potential therapeutic target for neurodegenerative diseases [9–12]. Existing evidences have suggested that AMPK signaling is also important for cell survival, especially under certain stress conditions [13, 14]. Here, we show that activation of AMPK is required ThIIA-mediated neuroprotection against OGDR.

## RESULTS

### Tanshinone IIA protects neuronal cells from oxygen glucose deprivation and re-oxygenation

**Figure 1:**
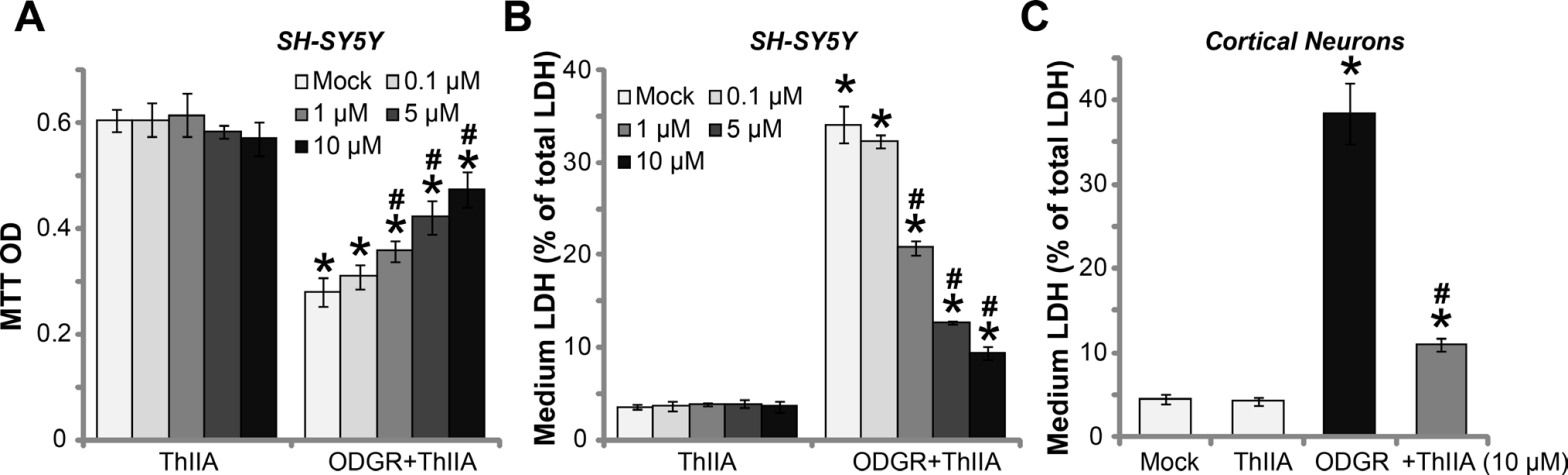
Tanshinone IIA protects neuronal cells from oxygen glucose deprivation and re-oxygenation SH-SY5Y neuronal cells (**A**–**B**) or the primary murine cortical neurons (**C**), pre-treated (for 30 min) with indicated concentration of Tanshinone IIA (“ThIIA”), were exposed to oxygen glucose deprivation (OGD, for 4 hours) and re-oxygenation (for 24 hours, ODGR), cell survival was tested by MTT assay (optic density, or OD at 550 nm was recorded, (A); Cell death was tested by LDH release (B and C). “Mock” stands for normoxia treatment (Same for all Figures). Data were presented as mean ± SD (*n* = 5). ^*^P < 0.05 *vs.* “Mock” group. ^#^*P* < 0.05 *vs.* ODGR only treatment (no ThIIA pre-treatment). Experiments in this figure were repeated four times, and similar results were obtained.

The current study aims to understand the potential effect of Tanshinone IIA (“ThIIA”) [15, 16] in neuron cells. SH-SY5Y is well-established human neuronal cell line [17–19]. The cell survival MTT assay results in Figure 1A showed that treatment with ThIIA alone at the tested concentrations (0.1–10 μM) failed to change SH-SY5Y cell survival. In line with previous findings [3], SH-SY5Y cells exposure to oxygen glucose deprivation (OGD) and re-oxygenation (ODGR) resulted in significant cell viability (“MTT optic density, OD) reduction [3], which was largely inhibited by pre-treatment of ThIIA (Figure 1A). ThIIA displayed a dose–dependent manner in protecting SH-SY5Y cells from OGDR (Figure 1A). OGDR-induced SH-SY5Y cell death was reflected by the increase of lactate dehydrogenase (LDH) release to the medium (Figure 1B). Pre-treatment with 1–10 μM of ThIIA significantly atte-nuated LDH release in OGDR-exposed SH-SY5Y cells (Figure 1B), again indicating its neuroprotective function. In the primary murine cortical neurons, OGDR exposure also resulted in dramatic cell death, causing LDH medium release (Figure 1C). Such effect was similarly inhibited by pre-treatment of ThIIA (10 μM) (Figure 1C). Treatment with the ThIIA alone failed to change LDH content in the neuronal cells (Figure 1B and 1C). Thus, ThIIA protects neuronal cells from OGDR.

### Tanshinone IIA inhibits OGDR-induced neuronal cell apoptosis

**Figure 2:**
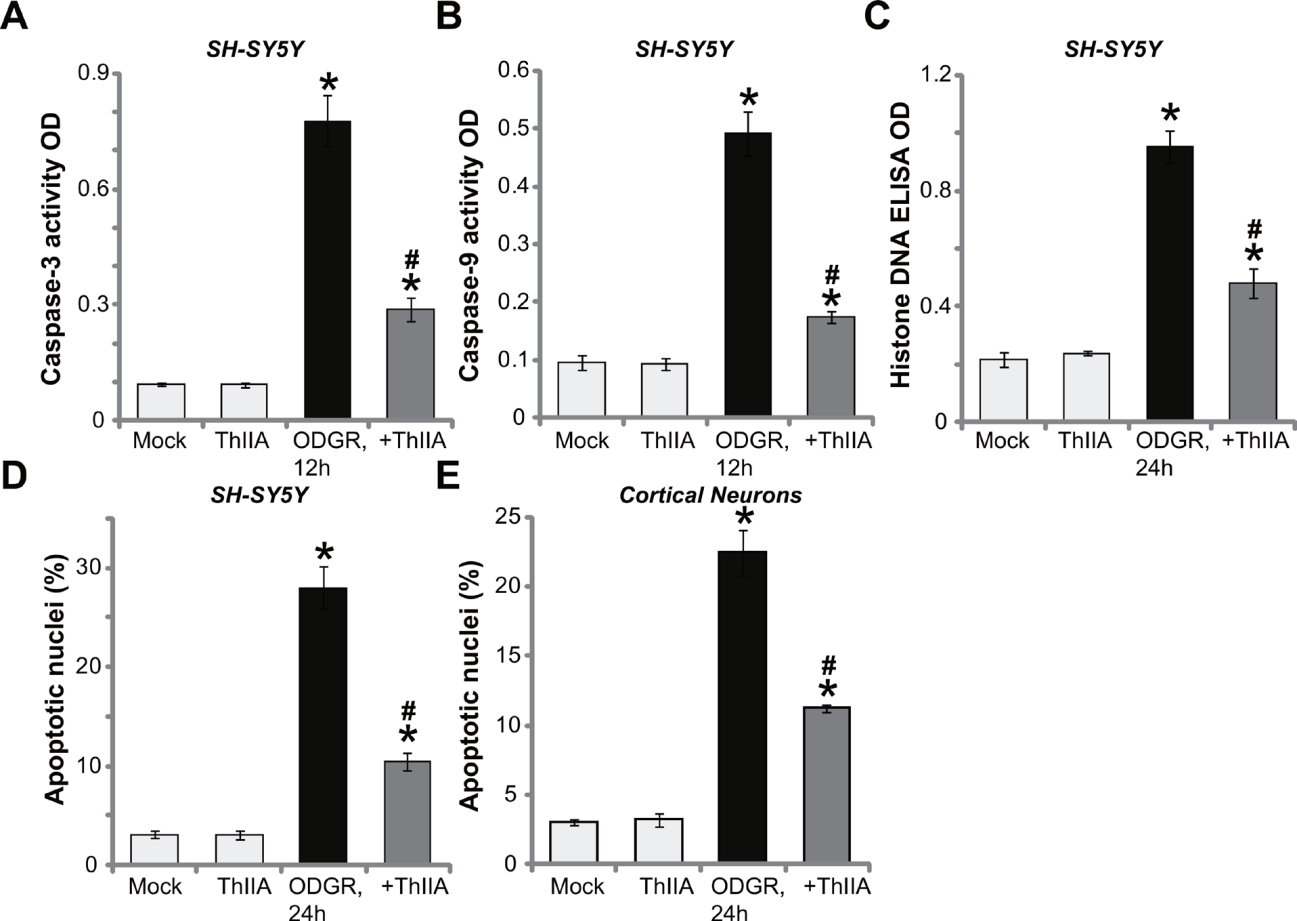
Tanshinone IIA inhibits OGDR-induced neuronal cell apoptosis SH-SY5Y neuronal cells (**A**–**D**) or the primary murine cortical neurons (**E**), pre-treated (for 30 min) with 10 μM of Tanshinone IIA (“ThIIA”), were exposed to oxygen glucose deprivation (OGD, for 4 hours) and re-oxygenation (for applied hours, ODGR), the cell apoptosis assays mentioned in the text were performed. Data were presented as mean ± SD (*n* = 5). ^*^P < 0.05 *vs.* “Mock” group. ^#^*P* < 0.05 *vs.* ODGR only treatment (no ThIIA pre-treatment). Experiments in this figure were repeated three times, and similar results were obtained.

The potential effect of ThIIA on neuronal cell apoptosis was studied. As demonstrated, SH-SY5Y cells with OGDR exposure presented with significant increased activity of both Caspase-3 (Figure 2A) and Caspase-9 (Figure 2B). Furthermore, the content of histone-bound DNA (an apoptosis marker) was also elevated in OGDR-treated SH-SY5Y cells (Figure 2C). These results suggested apoptosis activation after OGDR exposure (Figure 2A–2C). Significantly, pre-treatment with ThIIA (10 μM) largely attenuated OGDR-stimulated Caspase-3/-9 activation (Figure 2A and 2B) and histone-bound DNA increase (Figure 2C) in SH-SY5Y cells. To further study cell apoptosis, Hoechst 33342 staining assay was performed. The nuclei with condensed or fragmented Hoechst 33342 staining were labeled as the apoptotic nuclei [20, 21]. Its ratio was quantified. As shown in Figure 2D, OGDR dramatically increased the apoptosis ratio in SH-SY5Y cells, which was largely inhibited by ThIIA (10 μM) pretreatment (Figure 2D). The very similar results were also observed in the primary murine cortical neurons, where ThIIA (10 μM) pre-treatment efficiently suppressed OGDR-induced cell apoptosis (Hoechst assay, Figure 2E). It should be noted that treatment with ThIIA (10 μM) alone failed to induce apoptosis in the neuronal cells (Figure 2A–2E). These results demonstrate that ThIIA inhibits OGDR-induced neuronal cell apoptosis.

### ThIIA attenuates OGDR-induced mitochondrial depolarization, ROS production, lipid peroxidation and DNA damages

**Figure 3:**
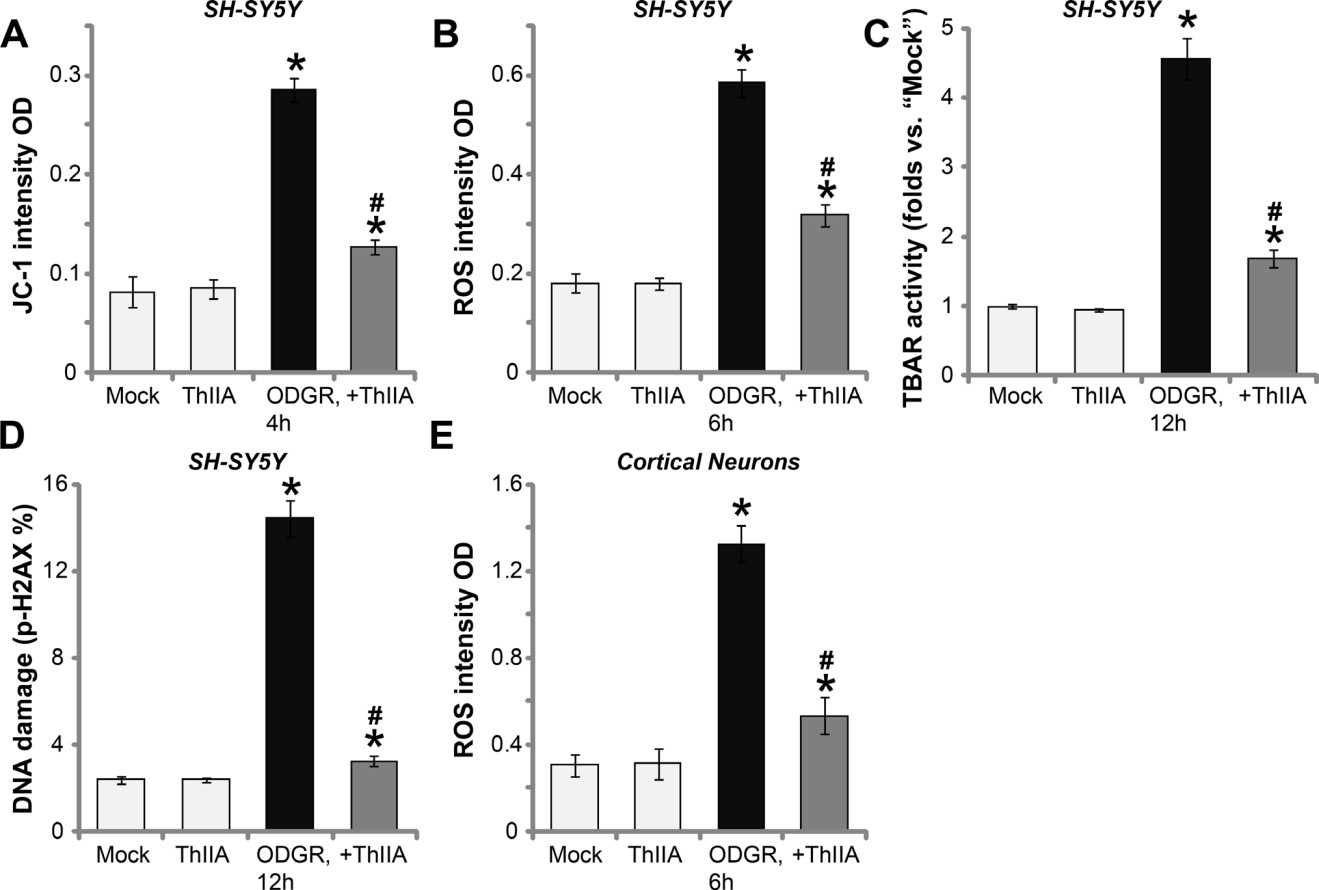
ThIIA attenuates OGDR-induced mitochondrial depolarization, ROS production, lipid peroxidation and DNA damages SH-SY5Y cells (**A**–**D**) or the primary murine cortical neurons (**E**), pre-treated (for 30 min) with 10 μM of Tanshinone IIA (“ThIIA”), were exposed to oxygen glucose deprivation (OGD, for 4 hours) and re-oxygenation (ODGR) for applied time, mitochondrial depolarization (A), ROS production (B and E), lipid peroxidation (C) and DNA damages (D) were tested by the assays mentioned in the text. Data were presented as mean ± SD (*n* = 5). ^*^P < 0.05 *vs.* “Mock” group. ^#^*P* < 0.05 *vs.* ODGR only treatment (no ThIIA pre-treatment). Experiments in this figure were repeated three times, and similar results were obtained.

Mechanism insight studies have revealed that OGDR to neuronal cells will be followed by mitochondrial dysfunction, swelling and depolarization, ROS production, which will lead to lipid peroxidation, DNA damages and eventually cell apoptosis [3, 22–24]. In line with these findings, we found that OGDR exposure in SH-SY5Y neuronal cells also induced mitochondrial depolarization and ROS production, which were tested by increase of JC-1 green fluorescence intensity (Figure 3A) and 2′,7′-dichlorofluorescein diacetate (DCFH-DA) fluorescence intensity (Figure 3B). Meanwhile, OGDR also caused lipid peroxidation (TBAR activity increase) (Figure 3C) and DNA damages (p-H2AX increase, Figure 3D). Remarkably, such effects by OGDR were dramatically attenuated with ThIIA (10 μM) pre-treatment (Figure 3A–3D). In the primary murine cortical neurons, ThIIA (10 μM) similarly inhibited OGDR-induced ROS production (Figure 3E). Treatment with ThIIA (10 μM) alone was ineffective (Figure 3A–3E).

### ThIIA activates AMPK signaling in neuronal cells

**Figure 4:**
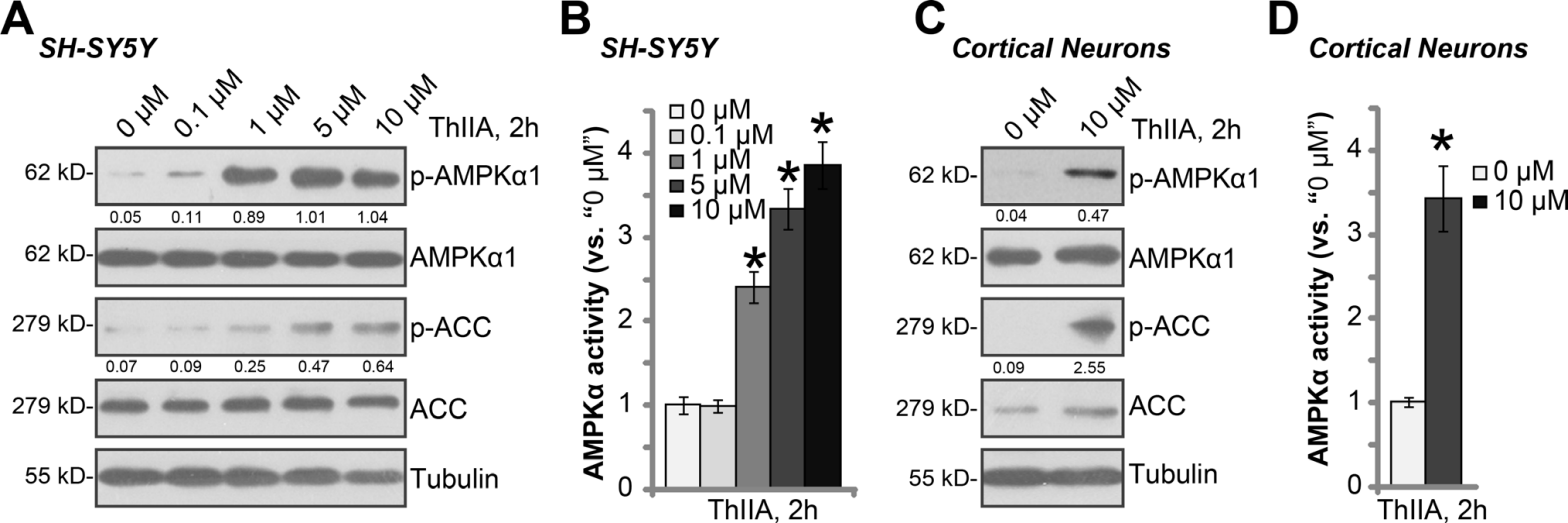
ThIIA activates AMPK signaling in neuronal cells SH-SY5Y neuronal cells (**A**–**B**) or the primary murine cortical neurons (**C**–**D**) were treated with applied concentration of Tanshinone IIA (“ThIIA”) for 2 hours, expressions of listed proteins in total cell lysates were shown (A and C); Relative AMPKα activity was also tested (B and D). AMPKα1/ACC phosphorylation was quantified (*vs.* total AMPKα1/ACC) (A and C). Data were presented as mean ± SD (*n* = 5). ^*^P < 0.05 *vs.* “0 μM” group (B and D). Experiments in this figure were repeated three times, and similar results were obtained.

As discussed, recent studies have suggested a pro-survival function of AMPK [25–27]. A number of AMPK activators were shown to protect cells from different stresses [28–31]. Previous studies have indicated that ThIIA could also activate AMPK signaling [32, 33]. We therefore examined AMPK signaling in ThIIA-treated neuronal cells. As shown in Figure 4A, in SH-SY5Y cells, ThIIA dose-dependently induced AMPK activation, which was reflected by increase of phosphorylations of AMPKα1 (at Thr-172) and its major downstream target acetyl-CoA carboxylase (ACC, at Ser-79). Meanwhile, AMPK activation was also evidenced by increase of AMPKα activity in ThIIA-treated SH-SY5Y cells (Figure 4B). The similar results were also obtained in ThIIA-treated primary murine cortical neurons, where AMPK/ACC phosphorylations (Figure 4C) and AMPKα activity (Figure 4D) were significantly boosted. These results imply that ThIIA activates AMPK signaling in neuronal cells.

### Activation of AMPK is required for ThIIA-induced neuroprotection

**Figure 5:**
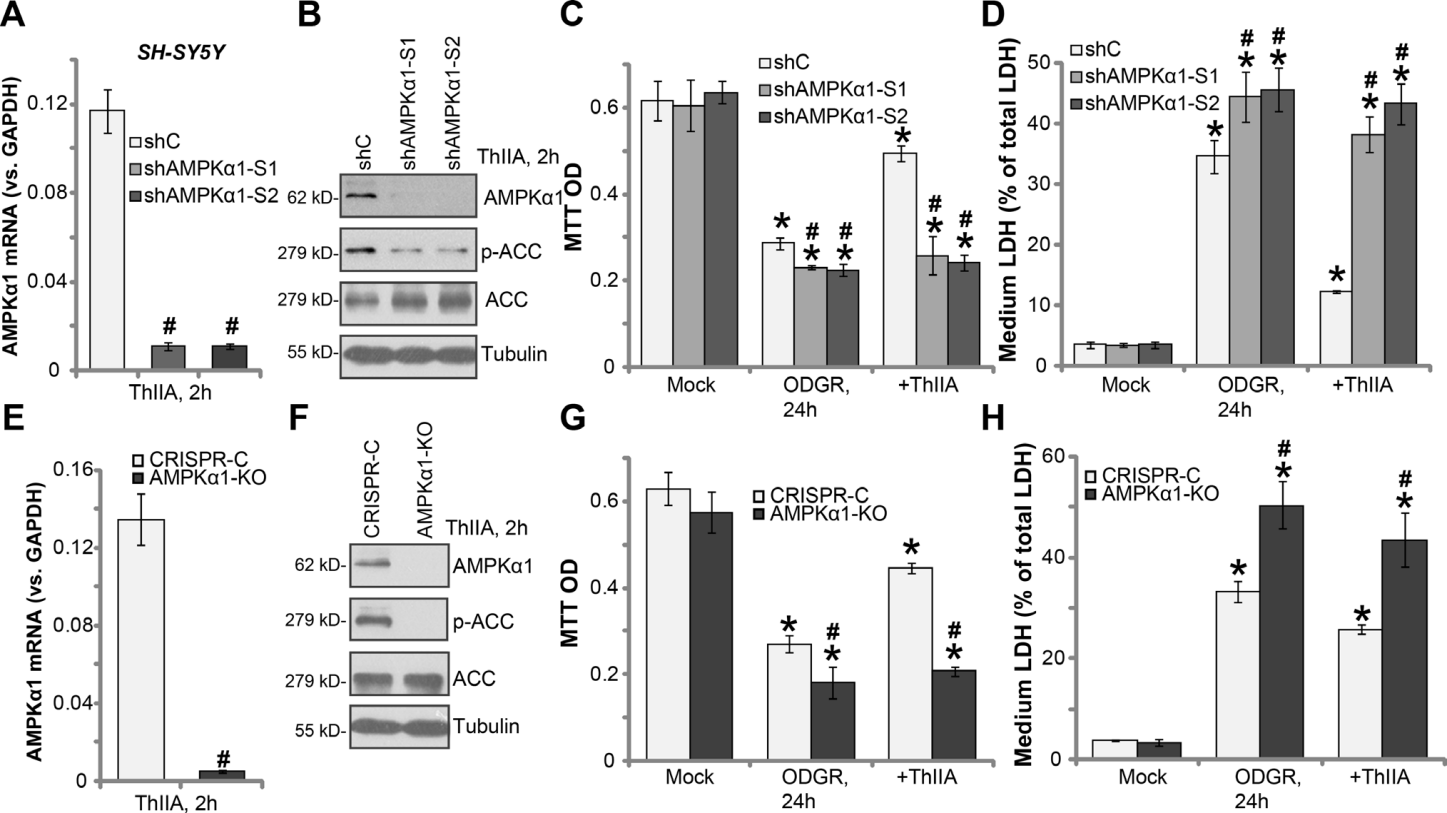
Activation of AMPK is required for ThIIA-induced neuroprotection The stable SH-SY5Y cells, expressing AMPKα1 shRNA (“S1/2”), or scramble control shRNA (“shC”) (**A**–**D**) as well as expressing CRISPR/Cas-9-AMPKα1 (“AMPKα1-KO”) or CRISPR/Cas-9 control (“CRISPR-C”), were pre-treated with 10 μM of Tanshinone IIA (“ThIIA”), with or without oxygen glucose deprivation (OGD, for 4 hours) and re-oxygenation (ODGR); *AMPKα1 mRNA* level was tested by qRT-PCR assay (A and **E**); Listed proteins in total cell lysates were shown (B and **F**); Cell survival and cell death were tested by MTT assay (C and **G**) and LDH release assay (D and **H**), respectively. Data were presented as mean ± SD (*n* = 5). ^*^P < 0.05 *vs.* “Mock” group. ^#^*P* < 0.05 *vs.* “shC” cells (C–D) or “CRISPR-C” cells (G and H). Experiments in this figure were repeated three times, and similar results were obtained.

In order to study the link between ThIIA-induced neuroprotection and AMPK activation, shRNA method was applied to knockdown AMPKα1. Two lentiviral AMPKα1 shRNAs, with non-overlapping sequences (S1 and S2, both from Dr. Lu's group [34, 35]), were added directly to cultured SH-SY5Y cells. Puromycin was then added to select stable cells. The quantitative real-time PCR (qRT-PCR) assay results confirmed that *AMPKα1 mRNA* level was dramatically downregulated in the stable SH-SY5Y cells with AMPKα1 shRNA (“S1/2”) (Figure 5A). Meanwhile, AMPKα1 protein expression and ThIIA-induced AMPK activation (reflected by p-ACC) were also largely inhibited (Figure 5B). The AMPKα1-silenced SH-SY5Y cells were more vulnerable to ODGR, showing increased viability reduction (Figure 5C) and LDH release (Figure 5D). Remarkably, ThIIA was almost ineffective in AMPKα1-silenced SH-SY5Y cells (Figure 5C and 5D). In the AMPKα1 shRNA SH-SY5Y cells, ThIIA treatment failed to protect against OGDR (Figure 5C and 5D).

The results above implied that activation of AMPK might be required for ThIIA-induced neuroprotection against OGDR. To further support our hypothesis, CRISPR/Cas-9 method was applied to completely knockout AMPKα1. The CRISPR/Cas-9-AMPKα1 vector was introduced to SH-SY5Y cells, and stable cells were again established by puromycin selection. qRT-PCR (Figure 5E) and Western blotting (Figure 5F) assay results confirmed complete depletion of AMPKα1 in the CRISPR/Cas-9-AMPKα1 (“AMPKα1-KO”) cells. ThIIA-induced ACC phosphorylation was completely blocked by CRISPR/Cas-9-AMPKα1 (Figure 5F). Similarly, OGDR exerted stronger cytotoxicity in AMPKα1-KO SH-SY5Y cells, as compared to cells with CRISPR/Cas-9 control (“CRISPR-C”) (Figure 5G and 5H). More importantly, ThIIA-mediated anti-OGDR neuroprotection was almost completely nullified in the AMPKα1-KO SH-SY5Y cells (Figure 5G and 5H). These results again confirm that activation of AMPK is required for ThIIA-induced neuroprotection.

### ThIIA increases miR-135b buts downregulates Ppm1e in neuronal cells

Very recent research efforts have characterized Ca^2+^/calmodulin-dependent protein kinase phosphatase (Ppm1e) as a key AMPKα1 phosphatase [31, 36, 37]. Ppm1e depletion or mutation could induce AMPKα1 phosphorylation and AMPK activation [31, 36, 37]. Here, the qRT-PCR assay results in Figure 6A demonstrated that ThIIA treatment caused a dramatic downregulation of *Ppm1e mRNA* in SH-SY5Y cells. Meanwhile, Ppm1e protein expression was also reduced by ThIIA (Figure 6B). On the other hand, the Ppm1e-targeting mRNA, miR-135b, was increased by ThIIA (Figure 6C). It should be noted that *Ppm1e mRNA* and protein expression as well as miR-135b expression were not changed by OGDR in SH-SY5Y cells (Figure 6A–6C).

The above results implied that ThIIA might possibly downregulate the AMPKα phosphatase Ppm1e to activate AMPK. Next, a miR-135b expressing construct (“miR135b-Vec”, a gift from Dr. Cui [31]) or the Ppm1e shRNA lentivirus (“sh-Ppm1e”, also from Dr. Cui [31]) was introduced to SH-SY5Y cells. As shown in Figure 6D, forced expression of miR-135b-Vec or the Ppm1e shRNA caused dramatic downregulation of *Ppm1e mRNA* in SH-SY5Y cells. Ppm1e protein expression was almost completed depleted in stable cells with miR135b-Vec or the shRNA (Figure 6E). Notably, miR-135b and Ppm1e shRNA both caused profound AMPK activation, which was evidenced by increased phosphorylations of AMPKα1 (Thr-172) and ACC (Ser-79) (Figure 6E). qRT-PCR assay results in Figure 6F confirmed increased miR-135b expression in cells with the miR135b-Vec, which was not changed by Ppm1e shRNA (Figure 6F).

Significantly, SH-SY5Y cells with miR-135b-Vec or the Ppm1e shRNA were largely protected from OGDR, presenting with decreased viability reduction (Figure 6G) and reduced LDH release following OGDR (Figure 6H). Therefore, activation of AMPK by miR-135b or Ppm1e shRNA also protected SH-SY5Y cells from OGDR (Figure 6G and 6H). Based on these results, we propose that ThIIA up-regulates miR-135b to downregulate Ppm1e, which possibly leads to AMPKα1 phosphorylation and AMPK activation, thus protecting neuronal cells from OGDR. Notably, miR-control vector (“miR-C”) plus the non-sense control shRNA lentivirus (“sh-C”) (“miR-C+sh-C”) didn't exert above activities in SH-SY5Y cells (Figure 6D–6H).

## DISCUSSION

Recent studies have verified a pivotal anti-oxidant function by activated AMPK. AMPK is important in maintaining NADPH balance [14]. Activated AMPK is shown to phosphorylate and inhibit ACC, causing decreased NADPH consumption [14]. Additionally, AMPK could also promote NADPH synthesis by fatty-acid oxidation [14]. Further, activated AMPK was also shown to activate Nrf2 signaling, a key anti-oxidant transcript factor [26, 38]. A recent study by Guo's group found that compound 13 (C13), an α1-selective AMPK activator, activated NADPH signaling to inhibit Dex-induced ROS production, therefore protecting osteoblastic cells [30]. Other AMPK activators showed similar anti-oxidant activity. Here, we showed that, in both SH-SY5Y neuronal cells and primary murine cortical neurons, ThIIA pre-treatment attenuated OGDR-induced viability reduction and apoptosis. Further, OGDR-induced mitochondrial depolarization, ROS production, lipid peroxidation and DNA damages were also attenuated by ThIIA in neuronal cells. ThIIA activated AMPK signaling, which was required for it-mediated neuroprotection against OGDR. AMPKα1 knockdown (by targeted shRNA) or complete knockout (by CRISPR-Cas-9 method) abolished ThIIA-induced AMPK activation and neuroprotection.

Phosphorylation on Thr172 of catalytic α1 subunit of AMPK is required for its activation [39, 40]. The kinase phosphorylation of Thr172 of AMPKα1 has been studied extensively [41]. Several AMPKα kinases have been indentified, including liver kinase B1 (LKB1) [42], CaMKK (calcium/calmodulin-dependent protein kinase kinase) [43] and TAK1 (Transforming growth factor-b-activated kinase 1) [44, 45]. Recent studies have focused on the phosphatases that are responsible for de-phosphorylating AMPKα1 at Thr172 [36]. Studies have indentified Ppm1e, a Ca^2+^/calmodulin-dependent protein kinase phosphatase [36], as well as PP2A catalytic subunit [29, 46], as possible AMPK phosphatases.

Inhibition or silence of the AMPK phosphatase has proven to be a fine strategy to induce AMPK activation. For instance, Cui's group demonstrated that miR-135b expression downregulated Ppm1e to activate AMPK signaling and protected osteoblastic cells [47]. microRNA-429-mediated silence of PP2A catalytic subunit, another AMPK phosphatase, also induced AMPK activation and protected cells from oxidative stress [29]. In the current study, we demonstrated that ThIIA upregulated miR-135b and downregulated the AMPK phosphatase Ppm1e, which possibly led to AMPKα1 phosphorylation and AMPK activation. This could also be the primary mechanism of ThIIA-mediated neuroprotection against OGDR. Indeed, knockdown of Ppm1e by targeted shRNA or forced expression of miR-135b also activated AMPK signaling and protected SH-SY5Y cells from OGDR, mimicking ThIIA functions.

## CONCLUSIONS

Together, we propose that AMPK activation by ThIIA protects neuronal cells from OGDR. miR-135b-mediated silence of Ppm1e could be the key mechanism of AMPK activation by ThIIA.

## METHODS

### Cell culture

Human neuronal cell line, SH-SY5Y, was purchased from the Cell Bank of Chinese Academy of Science (Shanghai, China). Cells were maintained in DMEM medium with 10% fetal bovine serum (FBS). Primary murine neurons were prepared from CA1 hippocampus of E14-E16 embryos of C57/B6 mouse. CA1 neurons (100,000 cells/cm^2^) were plated in serum-free neuron basal medium with 2% B27 supplement and 2 mM glutamine. All experimental procedures were approved by the Ethics Review Board and IACUC of authors’ institutions.

### Reagents and antibodies

Puromycin was purchased from Sigma (Shanghai, China). The Ppm1e antibody was from Dr. Cui's group [31]. All other antibodies utilized in this study were obtained from Cell Signaling Tech (Danvers, MA). The cell culture reagents were obtained from Gibco Life Technologies (Carlsbad, CA).

### OGD/re-oxygenation (OGDR)

Briefly, neuronal cells were first placed into an airtight chamber and equilibrated with a continuous flux of gas (95% N_2_/5% CO_2_). The chamber was sealed and placed in an incubator for 4 hours (mimic oxygen glucose deprivation). Afterwards, the neuronal cells returned back to the complete medium and re-oxygenated. “Mock” control cells were placed in norm-oxygenated complete medium.

### MTT assay

The survival of neuronal cells was evaluated by the routine 3-(4,5-dimethylthiazol-2-yl)-2,5-diphenylte-trazolium bromide (MTT, Sigma, Shanghai, China) according to the attached protocol. MTT optic density (OD) at 550 nm was recorded.

### LDH assay

LDH release to the conditional medium reflects cell death intensity, which was examined via a commercial available two-step LDH detection kit (Promega, Shanghai, China). LDH content in the conditional medium was normalized to the total LDH.

### Apoptosis assay by enzyme-linked immunosorbent assay (ELISA)

The Histone-DNA ELISA Detection Kit (Roche, Palo Alto, CA) was utilized to quantify cell apoptosis. Histone-DNA ELISA OD at 405 nm was recorded.

### Hoechst-33342 nuclei staining of apoptosis

Following the treatment, neuronal cells were stained with Hoechst-33342 (Sigma, Shanghai, China). Non-apoptotic nuclei were with faint delicate chromatin blue staining, and nuclei with intensified/fragmented Hoechst-33342 staining were labeled as apoptotic nuclei. Apoptotic nuclei ratio was calculated, from at least 200 cells of 6 random views (1: 100).

### Caspase activity assay

Following treatment, Caspase-3/-9 activity was tested via the Caspase-Glo-3/-9 activity assay kit (Promega, Nanjing, China), based on the attached protocols. The Caspase-3/-9 OD was recorded at 405 nm [48].

### Reactive oxygen species (ROS) detection

Neuronal cells were stained with 1 μM of DCFH-DA (Invitrogen, Shanghai, China) for 30 min. The DCF fluorescence signal was detected by a fluorescence microplate reader (Titertek Fluoroscan, Germany) at 550 nm.

### Mitochondrial depolarization assay

Briefly, neuronal cells were stained with JC-1 (10 μg/mL, Invitrogen, Shanghai, China) for 10 min at room temperature. JC-1 fluorescence intensity was examined using the fluorescence spectrofluorometer at 550 nm (Titertek Fluoroscan, Germany), testing membrane depolarization.

### Lipid peroxidation assay

As described [49], cellular lipid peroxidation level was evaluated using the routine thiobarbituric acid reactive substances (TBAR) assay [50]. 30 μg total cell lysates (per treatment) were mixed with 20% of acetic acid and thiobarbituric acid solution. After heating, the mixture was centrifuged, and the red pigment dye in the supernatant was examined by the microplate reader [50]. The TBAR activity, reflecting cellular lipid peroxidation level, was expressed as nM of malondialdehyde per mg protein.

### γ-H2AX assay

SH-SY5Y cells with the applied treatment were fixed and incubated with a mouse monoclonal anti-p-γ-H2AX antibody (Cell Signaling Tech, Shanghai, China), followed by adding a FITC-conjugated anti-mouse secondary antibody (Santa Cruz). SH-SY5Y cells were then subjected to FACS assay to determine p-γ-H2AX percentage, reflecting DNA damage intensity [51].

### Western blotting assay

The cell lysis buffer was purchased from Biyuntian (Wuxi, China). Quantified 30 μg of proteins from total cell lysates were separated by SDS-page gels (10–12%), which were transferred onto polyvinylidene difluoride (PVDF) blots (Millipore, Shanghai, China). After blocking, specific primary and corresponding secondary antibodies were added. The detection of the interested band was through the Enhanced chemiluminescence (ECL) reagents (Amersham Bioscience, Freiburg, Germany) and X-Ray film development. The ImageJ software was applied to quantify the intensity of each band.

### Quantitative real-time PCR

As previously reported [52–54], TRIzol reagents (Invitrogen, Shanghai, China) were utilized to extract total cellular RNA. Quantitative real time-PCR (“qRT-PCR”) assay was performed using the SYBR Green Master Mix Kit (Applied Biosystem) plus the ABI Prism 7600 Fast quantitative Real-Time PCR system (Foster City, CA). The *mRNA primers* for *Ppm1e*, *miR-135b-5p* and *GAPDH* were provided by Dr. Cui's group [31]. *mRNA primers* for *AMPK*α*1* were provided by Dr. Lu [34, 35]. Melt curve analysis was applied to calculate product melting temperature. GAPDH was tested as the internal reference gene. The 2^−ΔΔ*C*t^ method was applied for the quantification [52, 53].

### AMPKα activity assay

Total cell lysates were immunoprecipitated with anti-pan-AMPKα1 antibody (Cell Signaling Tech, Shanghai, China). The AMPKα activity was determined in kinase assay buffer [55] plus AMP-[γ-^32^P] ATP mixture, and SAMS peptide (HMRSAMSGLHLVKRR) [55]. The reaction was terminated by spotting the reaction mixture. The radioactivity was measured with scintillation counter.

### shRNA and selection of stable cells

The Ppm1e lentiviral shRNA was provided by Dr. Cui [31]. The shRNA lentivirus was added to SH-SY5Y cells for 24 hours. The stable SH-SY5Y cells were then selected by puromycin (1.0 μg/mL) for 8–10 days. The two lentivirus AMPKα1 shRNAs, with non-overlapping sequences (namely “S1” and “S2”), were provided by Dr. Lu's group [34, 35]. AMPKα1 shRNA lentivirus was added to SH-SY5Y cells, and stable cells were selected by puromycin. Expression of targeted protein in the stable cells was tested by Western blotting and/or qRT-PCR assays.

### CRISPR/Cas9-mediated knockout of AMPKα1

The small guide RNA (sgRNA) targeting human AMPKα1 was based on the Optimized CRISPR Design application (http://crispr.mit.edu/), which was provided by Genepharm (Shanghai, China). AMPKα1 sgRNA was then inserted into the lenti-CRISPR plasmid (Addgene, Shanghai, China), which was transfected to SH-SY5Y cells. Puromycin was added to select stable cells. Complete AMPKα1 knockout was verified by Western blotting assay.

### microRNA-135b expression

The lentiviral microRNA-135b (miR-135b) expression vector (0.15 μg construct per transfection), provided by Dr. Cui [31], was transfected to SH-SY5Y cells by Lipofectamine 2000 (Invitrogen, Shanghai, China). The stable SH-SY5Y cells were then selected by puromycin (1.0 μg/mL) for 8-10 days. Expression of miRNA-135b-5p in above cells was verified using qRT-PCR assay.

### Statistical analysis

Data were presented as mean ± standard deviation (SD). Statistics were analyzed by one-way ANOVA followed by a Scheffe’ and Tukey Test (SPSS 16.0, Chicago, CA). *P* < 0.05 means significant difference.
